# Evaluation of the Stress Level of Children with Idiopathic Scoliosis in relation to the Method of Treatment and Parameters of the Deformity

**DOI:** 10.1100/2012/538409

**Published:** 2012-07-31

**Authors:** Justyna Leszczewska, Dariusz Czaprowski, Paulina Pawłowska, Aleksandra Kolwicz, Tomasz Kotwicki

**Affiliations:** ^1^Department of Physiotherapy, Józef Rusiecki University College in Olsztyn, 10-243 Olsztyn, Poland; ^2^Center for Body Posture of the Health and Sports Center Foundation, 10-243 Olsztyn, Poland; ^3^Rehasport Clinic, 60-201 Poznań, Poland; ^4^Department of Pediatric Orthopedics and Traumatology, Poznań University of Medical Sciences, 60-512 Poznań, Poland

## Abstract

Stress level due to existing body deformity as well as to the treatment with a corrective brace is one of factors influencing the quality of life of children with idiopathic scoliosis undergoing non-surgical management. The purpose of the study was to evaluate the stress level among children suffering from idiopathic scoliosis in relation to the method of treatment and the parameters of the deformity. Seventy-three patients with idiopathic scoliosis participated in the study. Fifty-two children were treated by means of physiotherapy, while 21 patients were treated with both Cheneau corrective brace and physiotherapy. To assess the stress level related to the deformity itself and to the method of treatment with corrective brace, the two Bad Sobernheim Stress Questionnaires (BSSQs) were applied, the BSSQ Deformity and the BSSQ Brace, respectively.

## 1. Introduction

One of factors influencing the quality of life is the level of stress that accompanies a particular dysfunction. It affects not only the patients themselves but also their families [1–5]. Considering the number of questionnaires estimating the quality of life, various aspects of the influence of the disease and its treatment on the quality of life can be evaluated in a simple and objective manner. Some of the questionnaires, such as QLPSD (Quality of Life Profile for Spine Deformities), BSSQ (Bad Sobernheim Stress Questionnaire), and SRS-22 HRQL (Scoliosis Research Society Health-Related Quality of Life Questionnaire for Idiopathic Scoliosis), are specific to patients suffering from idiopathic scoliosis (IS). 

In the case of IS, the deformities and asymmetries in body build, decreased physical fitness, and the form of therapy represent the stress-creating factors [6–8]. Treatment of IS includes physiotherapy, bracing, or surgical intervention [9]. Irrespective, the form, the treatment frequently requires change in the lifestyle [3, 7]. The patients' awareness, patience, self-discipline, and approval for therapy are essential elements in effective and successful treatment [9].

The aim of the study was to evaluate the stress level among children suffering from idiopathic scoliosis, depending on the method of treatment and the parameters of the deformity.

## 2. Material and Methods 

### 2.1. Study Group

The inclusion criteria were idiopathic scoliosis diagnosed on the basis of the X-ray, age 9 to 18 years, and conservative treatment in progress (physiotherapy or Cheneau brace combined with physiotherapy). The patients who had formerly received surgical treatment for scoliosis were excluded. Official approval had been granted by the local Ethics Committee. The researchers received written consent from parents and children.

The analysis was performed in the group of 73 patients (64 girls, 9 boys). Single-curve, right thoracic scoliosis was diagnosed in 41 patients, whereas double-curve scoliosis (right thoracic and left lumbar) in 32. Fifty-two children were treated by means of physiotherapy, whilst 21 children were treated with both Cheneau brace and physiotherapy. Both the subgroup treated with physiotherapy and the subgroup treated with Cheneau brace and physiotherapy were matched for age, height, weight, BMI, and the length of scoliosis (*P* > 0.05). Significant differences between the subgroups were found in the severity of the curvature according to Cobb (*P* = 0.001) and in the apical vertebra rotation (*P* = 0.006), the physiotherapy subgroup representing lover values. The patients were asked about the brace wearing time (number of hours per day) and their engagement in physical activities (number of hours per week) (Table 1). 

In order to assess the stress level, the BSSQ Deformity and the BSSQ Brace Questionnaires were applied. Both questionnaires were validated to Polish [11]. 

### 2.2. BSSQ Questionnaires

Each of the two BSSQ questionnaires (the BSSQ Brace and the BSSQ Deformity) consists of eight closed questions with four possible answers. In the BSSQ-Deformity, the questions concern the impact of spinal deformity on mood, social interactions, and, in consequence, on the level of stress, whereas the BSSQ-Brace questionnaire focuses on psychological strain due to conservative treatment and evaluates the manner in which wearing the brace influences mood, social interactions, and, in consequence, the level of stress. Both questionnaires are characterized by high internal consistency, which makes them credible tools in evaluating the mental state of patients [6, 11, 12].

### 2.3. Interpretation of the Questionnaire

The minimum score in each questionnaire is 0, indicating the highest level of stress, whereas the maximum score is 24 (the lowest stress level). Botens-Helmus et al. proposed the range 0–8 (strong stress), 9–16 (medium stress), and 17–24 (little stress) [6].

### 2.4. Procedure

52 patients (71%) treated exclusively by means of physiotherapy completed the BSSQ-Deformity questionnaire, and 21 patients (29%) undergoing conservative treatment with Cheneau brace and physiotherapy completed two questionnaires (the BSSQ-Deformity and the BSSQ-Brace).

In order to determine the level of stress the following analysis was performed: (1) comparing the results of the BSSQ-Deformity of the subgroup of children treated by means of physiotherapy with the subgroup treated by both brace and physiotherapy, (2) comparing the result of the BSSQ-Deformity in patients with single-curve scoliosis versus the patients having double-curve scoliosis, (3) establishing the correlation of the results obtained with BSSQ-Deformity versus the scoliosis angle value, the length of scoliosis, and the apical vertebral rotation in the thoracic and lumbar spine, respectively, (4) establishing the correlation between the result of the BSSQ-Brace and the brace wearing time per day, as well as (5) establishing the correlation between the result of the BSSQ-Deformity and physical activity of the subjects. 

### 2.5. Statistical Analysis

The results underwent statistical analysis with Statistica 8.0 (StatSoft, USA). Distribution was assessed with Kolmogorov-Smirnov test. Student's, Mann-Whitney *U* was used as well as Pearson's and Spearman's correlation of ranks. A value of *P* < 0.05 was adopted as the significance level.

## 3. Results 

The median score obtained with the BSSQ-Deformity questionnaire in the subgroup of children treated solely by means of physiotherapy was 21, while in the subgroup wearing the brace it was 19; these results indicate little stress. There was no significant difference (*P* = 0.31) between the medians. There was no difference (*P* = 0.33) between children with single-curve versus those with double-curve scoliosis (Table 2). 

The median score of the BSSQ-Brace questionnaire was 10, which indicates medium stress. The correlation between the results of the two BSSQ questionnaires and the parameters of scoliosis, the time of brace wearing, and the physical activity is shown in Table 3.

## 4. Discussion

The methods of treatment of idiopathic scoliosis involve physiotherapy, corrective braces, and surgical procedures [9]. The value of conservative therapy is disputable; however the effectiveness of braces in reducing the rate of progression of scoliosis has been confirmed [9, 13–15]. Reports have indicated negative impact of brace treatment on the patient's mental state, self-image, and social interactions [3, 6, 16–18]. 

In the present study the authors assessed not only the relationship of stress level to the method of the treatment but also to the parameters of deformity and physical activity which had not been previously investigated.

Despite the fact that the children treated by both brace and physiotherapy presented significantly more severe Cobb angle value than children treated only by physiotherapy, there was no significant difference regarding the BSSQ-Deformity median scores obtained in both subgroups. Similar results were presented by Kotwicki et al. [8], Misterska et al. [11], as well as Kinel et al. [19]. Ólafsson et al. stated that bracing does not exert negative influence on self-esteem [20]. 

**Figure 1:**
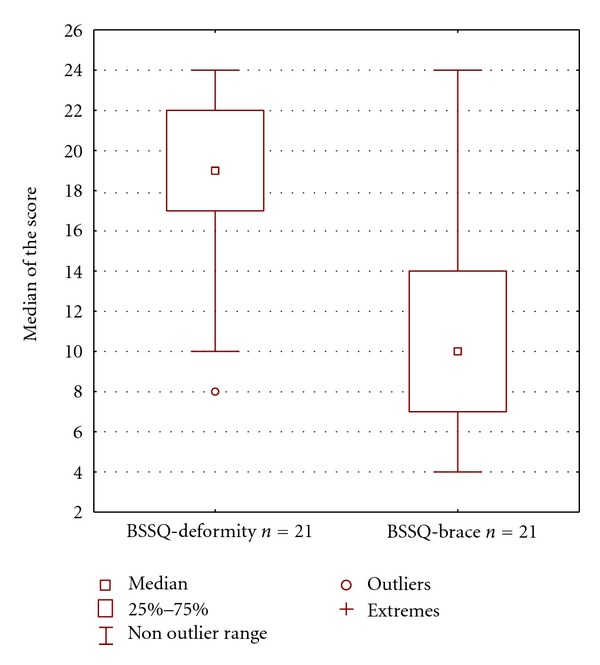
The scores of two questionnaires: the BSSQ-Deformity and the BSSQ-Brace applied to the same subgroup of patients treated with both brace and physiotherapy (*n* = 21).

On the other hand in the present study the authors observed that the patients managed with brace and physiotherapy revealed lower score with BSSQ-Brace (median = 10) comparing to BSSQ-Deformity (median = 19) (Figure 1). This suggests that the method of therapy, not the fact of having the deformity itself, has the dominant influence on the stress level.

According to Ugwonali et al., the brace treatment causes a minimum influence on the quality of life of patients with adolescent idiopathic scoliosis [21]. In opposite, the results obtained by Pham et al. [3], Botens-Helmus et al. [6], Cheung et al. [16], Sapountzi-Krepia et al. [17], and Reichel and Schanz [18] confirm negative impact of brace on the quality of life. The authors are of the opinion that divergent results are caused by the use of different questionnaires. Furthermore, some studies focus on the quality of life of patients during treatment, whereas others take into consideration different periods after its completion [14, 22]. Some researchers suggest that decrease of the quality of life is a temporary phenomenon. Once the treatment completed, one may expect improvement [2, 19, 21, 23]. 

According to Misterska et al., the level of stress has no significant relation with the brace wearing time per day [11]. However, Pham et al. compared the quality of life in three groups of patients, one of which wore the brace twenty-four hours a day, the second one wore the brace at night, and the third one did not use the brace whatsoever. Their results indicate that the quality of life was the lowest among the patients wearing the brace twenty-four hours a day and the highest among the patients who were not brace treated [3]. Our findings showed that there was no significant correlation between brace wearing time and the score of the BSSQ Brace questionnaire. These observations are in agreement with results of Misterska et al. [11]. 

This study has shown a significant negative correlation between the level of stress and the angle value of curvature, which is in agreement with the results by Kotwicki et al. and Kinel et al. [8, 19]. Cheung et al. used the SRS-22 questionnaire to estimate the quality of life of patients with idiopathic scoliosis during brace treatment and discovered a relationship between the quality of life and the Cobb angle value, when the angle value of curvature did not exceed 20° [16]. Misterska et al. [11] as well as Botens-Helmus et al. [6] did not observe such relation despite the fact that they used the same tool (BSSQ). Bunge et al. observed no significant correlation between quality of life and Cobb angle value; their research involved patients after surgical treatment and after the completion of brace treatment [23].

The correlation of the level of stress with the angle of vertebral rotation in the lumbar spine was significant, possibly caused by the fact that the rotation of vertebrae in the lumbar region may influence the asymmetries within the hips and flanks [24]. It is an interesting fact that the vertebral rotation in the thoracic region had no relation with the stress level, probably due to the localization to be less visible for patients themselves and easier to conceal. Similar results were presented by Misterska et al., who demonstrated that the level of stress increases along with the value of the rotation angle measured by the Perdiolle method [11]. The measurement of the angle of trunk rotation with the Bunnell method for the primary and compensatory curvature also showed a significant correlation with the quality of life, which was reported by Kotwicki et al. [8] and Kinel et al. [19]. According to results of the present study the number of curvatures of scoliosis and its length did not influence the level of stress.

The authors have not found reports which would determine the relationship between the stress level in case of idiopathic scoliosis conservatively treated and the level of physical activity. The findings have confirmed that the level of stress decreases along with an increase of physical activity. 

## 5. Conclusions 

Regarding deformity both the children treated only with physiotherapy and the children treated with brace and physiotherapy presented a little stress. The result of the BSSQ-Brace indicated medium stress of children treated with brace. The main factor creating stress seemed to be wearing the brace rather than having the deformity itself. The stress level was related to the scoliosis angle value and the angle of apical vertebra rotation in the lumbar spine. The number of curvatures, the length of scoliosis, and the angle of vertebral rotation in the thoracic spine were not related with the stress level. Physical activity was a factor reducing stress in patients with idiopathic scoliosis.

## Figures and Tables

**Table 1:** The characteristics of the study group (*n* = 73).

Variable	Average	SD	Min	Max
Age (years)	13.9	2.1	9	18
Height (m)	1.61	0.09	1.34	1.90
Weight (kg)	50.2	12.3	29.0	91.0
BMI (kg m^−2^)	19.2	3.7	14.0	31.1
Cobb angle (^°^)	23.9	17.7	11	103
AVR	1.74	0.9	1	4
Length of scoliosis (number of vertebrae)	8.8	3.1	3	15
Brace wearing time (hours/day)	19.3	4.6	8	24
Physical activity (hours/week)	4.1	2.3	0	11

BMI: body mass index, AVR: apical vertebra rotation quantified according to Cobb method [10].

**Table 2:** Medians of the BSSQ-Deformity in two subgroups of patients depending on the method of treatment and the number of curvatures of scoliosis.

Questionnaire	*n*	Patients subgroup	Median	Range	*P*
BSSQ Deformity	73	Physiotherapy (*n* = 52)	21	6–24	0.31
		Brace and physiotherapy (*n* = 21)	19	8–24	
BSSQ Deformity	73	Single-curve scoliosis (*n* = 41)	21	10–24	0.33
		Double-curve scoliosis (*n* = 32)	19	6–24	

**Table 3:** The correlation of the BSSQs scores with the parameters of scoliosis, the time of brace wearing, and the physical activity.

	BSSQ deformity (*n* = 73)	BSSQ brace (*n* = 21)
Angle of curvature	*r* = −0.32; *P* = 0.007	—
Length of curvature (number of vertebrae)	*r* = −0.11; *P* = 0.38	—
Thoracic apical vertebra rotation	*r* = 0.28; *P* = 0.33	—
Lumbar apical vertebra rotation	*r* = −0.66; *P* = 0.002	—
Brace wearing time (hours/day)	—	*r* = 0.13; *P* = 0.57
Physical activity (hours/week)	*r* = 0.24; *P* = 0.04	—

*r*: The coefficient of correlation, *P*: Level of significance.
